# Among the Colleges

**Published:** 1897-11

**Authors:** 


### AMONG THE COLLEGES.

---

### ONTARIO VETERINARY COLLEGE, LIMITED.

Dr. J. A. Amyot will this year instruct the students in animal physiology in place of Dr. G. A. Peters of last year. Dr. D. K. Smith, who is one of the '97 graduates from this college, will occupy the chair of practical microscopy. The first and second prizes have each been reduced $5 in value. The college has this year affiliated with the Toronto University, and will probably change its course of instruction and length of term at the commencement of its next session of 1898 and 1899.

The college is said to have over one hundred students in its classes for 1897 and 1898; some seventeen of the United States are represented.

